# Colistin Interaction and Surface Changes Associated with *mcr-*1 Conferred Plasmid Mediated Resistance in *E. coli* and *A. veronii* Strains

**DOI:** 10.3390/pharmaceutics14020295

**Published:** 2022-01-27

**Authors:** Firdoos Ahmad Gogry, Mohammad Tahir Siddiqui, Insha Sultan, Fohad Mabood Husain, Abdulaziz A. Al-Kheraif, Asghar Ali, Qazi Mohd. Rizwanul Haq

**Affiliations:** 1 Department of Biosciences, Jamia Millia Islamia, New Delhi 110025, India; firdous071@gmail.com (F.A.G.); tahir.siddiqui1709@gmail.com (M.T.S.); inshasultan12@gmail.com (I.S.); asg.bstlko@gmail.com (A.A.); 2 Department of Food Science and Nutrition, College of Food and Agriculture Sciences, King Saud University, Riyadh 11451, Saudi Arabia; fhusain@ksu.edu.sa; 3 Dental Biomaterials Research Chair, Dental Health Department, College of Applied Medical Sciences, King Saud University, Riyadh 11451, Saudi Arabia; aalkhuraif@ksu.edu.sa

**Keywords:** Gram-negative bacteria, lipopolysaccharide, colistin resistance, *mcr*-1, electrostatic interaction

## Abstract

Colistin, a polycationic antimicrobial peptide, is one of the last-resort antibiotics for treating infections caused by carbapenem-resistant Gram-negative bacteria. The antibacterial activity of colistin occurs through electrostatic interaction between the polycationic peptide group of colistin and the negatively charged phosphate groups of lipid A membrane. This study investigated the interaction of colistin with the outer membrane and surface constituents of resistant and susceptible strains of *Escherichia coli* and *Aeromonas veronii* harboring *mcr-*1 resistance gene. Bacterial membrane and lipopolysaccharide used in this study were isolated from susceptible as well as colistin-resistant strains of *E. coli* and *A. veronii*. Interaction of colistin with the bacterial surface was studied by deoxycholate and lysozyme sensitivity test, N-phenyl-1-naphthylamine (NPN) uptake assay, Atomic force microscopy (AFM), Zeta potential measurements and ^1^H NMR. The binding affinity of colistin was found to be lower with outer membrane from resistant strains in comparison with the susceptible strains. Colistin exposure enhances the outer membrane permeability of the susceptible strains to deoxycholate and lysozyme. However, on the other hand, colistin dose of 256 µg/mL did not permeabilize the outer membrane of resistant bacteria. The NPN permeability in resistant strains was greater in comparison with susceptible strains. Atomic force microscopy images depicted smooth, featherless and deformed membranes in treated susceptible cells. Contrary to the above, resistant treated cells displayed surface roughness topography even at 256 µg/mL colistin concentration. Surface charge alterations were confirmed by Zeta potential measurements as a function of the growth phase. Mid-logarithmic phase susceptible strains showed a greater negative charge than resistant strains upon exposure to colistin. However, there was no statistical variation in the Zeta potential measurements between resistant and susceptible strains at the stationary phase. NMR analysis revealed line broadening in susceptible strains with increasing colistin: LPS aggregates mass ratio. Moreover, resistant strains did not show line broadening for the outer membrane, even at the highest mass ratio. The findings of this study suggest that the resistant strains of *E. coli* and *A. veronii* can block the electrostatic contact between the cationic peptide and anionic lipid A component that drives the first phase of colistin action, thereby preventing hydrophobically driven second-tier action of colistin on the outer lipopolysaccharide layer.

## 1. Introduction

The steady increase in antibiotic resistance coupled with a decline in the development of new drugs, is leading the world towards the pre-antibiotic era [1,2]. This global public health threat requires immediate multidisciplinary steps to achieve sustainable development goals (SDGs) [3]. New antibiotics active against Gram-positive bacteria provided respite to some extent [4], but infections caused by antibiotic-resistant Gram-negative bacteria are emerging as a greater threat. The complex structure of the cell envelope in Gram-negative bacteria presents a permeability barrier for the effective passage of several antibiotics. This barrier is attributed to negatively charged lipopolysaccharide molecules. The negatively charged dense surface layer is favorable to the action of antibiotics. Polymyxins are antibiotics, structurally comprised of a cyclic heptapeptide with five major chemical compounds viz: polymyxin A, B, C, D and E. These compounds are differentiated based on variation in their amino acid sequences and fatty acid side chains. The prime representatives of polymyxin that have been used in clinical practice are polymyxin E (colistin) and polymyxin B [5,6,7]. Colistin is a polypeptide antibiotic isolated in 1947 from the bacterium *Paenibacillus polymyxa* subsp. *Colistinus* [8,9]. Failure of carbapenems against Gram-negative bacteria has led to the unprecedented increase in the use of colistin (one of the last resort drugs) and subsequent emergence and dissemination of colistin resistance [5]. Resistance to polymyxins has mainly emerged against polymyxin E class (colistin), a cationic polypeptide drug, composed of a cyclic decapeptide attached by an amide linkage to a fatty acyl chain which is differentiated by single amino acid from polymyxin B compound [7,10]. Colistin exerts its activity on Gram-negative bacteria through two-step mechanisms that are initial binding and employ permeabilization of the outer membrane, subsequently destabilizing the cytoplasmic membrane. The vital step in the action of colistin is the electrostatic interaction between the cationic peptide and anionic lipid A, the endotoxin component of lipopolysaccharide [9].

Colistin resistance usually involves modulation of lipid A that decreases or removes early charge-based interaction with colistin through up-regulation of multi-step capsular polysaccharide expression. Two-component regulatory systems (PmrAB and PhoPQ), are needed for the modulation of lipid A with palmitoyl and 4-amino-4-deoxy-L-arabinopyranose (Arap4N) addition. PmrA-PmrB system is negatively controlled by the regulator mgrB gene [11,12]. Therefore, a mutation in the form of insertion or frameshift on mgrB leads to the upregulation of two-component systems which ultimately causes modification of the outer membrane hence the emergence of colistin resistance. Moreover, bacterial species like *Acinetobacter baumannii* also contain mutations within genes (either lpxA, lpxC, or lpxD) essential for lipid A biosynthesis and lose the ability to produce lipid A and consequently lipopolysaccharide chain [10]. Liu et al., first reported plasmid-mediated colistin resistance *mcr*-1 gene from China [5], thereafter, several studies have reported variants of *mcr* genes determining colistin resistance [13,14,15]. The emergence, structure and mechanism in bacteria from animal and human isolates present evidence for the dissemination of *mcr*-1 from veterinary to human beings [5]. The *mcr*-1 has been identified in bacterial isolates due to the continuous use of colistin in human and veterinary purposes [16,17,18]. There is a clear indication of rapid dissemination of colistin resistance which requires further studies to evaluate the factors involved, mechanism of acquisition and dissemination.

Resistance to colistin is well characterized at the genetic level, but still, there is a deficit of information related to the various surface properties of resistant bacteria that physically obstruct colistin from initial binding and exert bactericidal activity. In this study, we have used a series of biophysical and biochemical parameters to examine the correlation between colistin resistance and the ability of colistin drug to bind with the bacterial outer membrane isolated from paired susceptible *E. coli* ATCC 25922 and *A. veronii* ATCC 35624, and colistin-resistant strains of *E. coli* and *A. veronii*. The obtained data will reveal the association between bactericidal activity and the binding of colistin drug with the biophysical barriers and other membrane components of the *E. coli* and *A. veronii* surfaces that confer resistance.

## 2. Methodology

### 2.1. Materials

Colistin Sulfate, lysozyme, deoxycholate, N-phenyl-1-naphthylamine (NPN), NMR tubes and D_2_O were obtained from Sigma-Aldrich (St. Louis, MO, USA). The high purity commercially available reagents were used for protocols. Colistin stock solutions (10 mg/mL) in Milli-Q water were prepared and all prepared solutions were stored at 4 °C.

### 2.2. Bacterial Strains and Growth Conditions

Colistin-resistant strains of *E. coli*(AF15) and *A. veronii* (AF6) (both *mcr*-1 positive) were selected from the previous study [19] and *E. coli* ATCC 25922 and *A. veronii* ATCC 35624 were used as a negative reference strain. The molecular analysis of *mcr*-1 positive bacterial isolates understudy have been sequenced and interpreted. The plasmid profiling confirmed the association of *mcr*-1 in IncF plasmid in AF15 and IncX plasmid in AF6. The genetic characteristics were submitted in the NCBI database with accession no. MN367313 and MN367312 for AF6 and AF15, respectively. Bacterial cultures were initially inoculated in 5 mL of cation-adjusted Mueller–Hinton broth (CaMHB; Himedia), from which a 1 in 100 dilution was performed in fresh broth to obtain mid-logarithmic cultures according to the OD at 500 nm (OD_500 nm_  =  0.4–0.6). All resistant and susceptible broth cultures were incubated at 37 °C in an incubator shaker (180 rpm)

### 2.3. Determination of Minimum Inhibitory Concentration (MIC)

MIC for each strain was determined by the broth microdilution protocol and interpreted by the European Committee on Antimicrobial Susceptibility Testing guidelines [20]. All experiments were performed in polypropylene 96-well plates having CaMHB media. 100 μL of bacterial suspension were inoculated in microtiter wells with increasing colistin (0–1024 μg/mL) concentrations with two-fold dilution in the 96-well plates. The MICs were explained as the lowest concentration at which visible growth of bacterial cells was inhibited with continuous 15–18 h incubation at 37 °C.

### 2.4. LPS Extraction

Bacteria for LPS extraction were grown in Luria Broth overnight with continuous shaking (37 °C, 180 rpm). The growth media was supplemented with 2 μg/mL colistin. OD_600_ of the culture were recorded in a spectrophotometer (Thermo Scientific MultiskanGo, Waltham, MA, USA). The extraction of LPS was carried out following the hot/phenol protocol described earlier with minor modification [21,22]:

(i) 1.5 mL suspension of bacterial culture (OD_600_ of 0.5) was pelleted at 8000 (rpm) for 10 min, supernatants were discarded and pellets suspended in 200 μL of 1X SDS-buffer.
(ii) Resuspended samples were boiled for 15 min in a water bath and left to cool for 15 min at room temperature.
(iii) The samples were treated with DNase I, RNase and Proteinase K. For DNase and RNase treatment 5 μL of each (10 mg/mL) were added and incubated at 37 °C for 30 min. Each sample was further treated with 10 μL Proteinase K (10 mg/mL) for 3 h at 59 °C.
(iv) After proteinase K treatment, 200 μL of ice-cold Tris-saturated phenol was added to each sample and vortexed for 5 to 10 s followed by 15 min incubation at 65 °C.
(v) Thereafter, 1 mL of diethyl ether was added to each prepared sample, vortexed and centrifuged at 15,000× *g* (rpm) for 10 min. Tubes were carefully removed from the centrifuge and the bottom blue layer was transferred to a fresh tube. This step (v) was repeated thrice.
(vi) 200 μL of 2X SDS buffer was added to each sample and run on 12% SDS-PAGE. Fifteen μL of LPS preparation was sufficient to visualize discrete bands on the gel.

### 2.5. Deoxycholate Sensitivity Assay

Mid-logarithmic phase bacterial cells (OD_500nm_ = 0.4–0.6) were centrifuged at 4000× *g* (rpm) for 5 min. Pelleted cells were re-suspended in 5 mM (5 mL) HEPES buffer (pH 7.2), aliquots were supplemented with colistin in order of increasing concentration followed by incubation for 10 min at 37 °C. After incubation ODs were measured (OD_500nm_ = 0.4–0.6) and pelleted cells were re-suspended in 5 mM (5 mL) of HEPES buffer having 0.25% (*w*/*v*) deoxycholate. These treated bacterial suspensions were incubated for 10 min at 37 °C temperature and a decline in OD_500 nm_ was subsequently recorded as previously described [23]. Results were illustrated in the form of OD of every sample in the absence of deoxycholate.

### 2.6. Lysozyme Sensitivity Assay

Mid-logarithmic phase resistant and susceptible bacterial cell suspensions (OD_500 nm_ = 0.4–0.6) were treated with Colistin (9 μg/mL) and Lysozyme (5 μg/mL) and a decrease in OD reading was recorded. The obtained data were interpreted with control reaction OD in absence of lysozyme [23].

### 2.7. N-phenyl-1-Naphthylamine Uptake Assay

NPN uptake measurements were done as earlier described [24]. Mid-logarithmic phase resistant and susceptible cells were pelleted and again resuspended in 5 mM HEPES buffer (pH 7.2) dissolved in 1 mM sodium azide. This step was repeated thrice and OD_500 nm_ of final suspension was adjusted to 0.5. The cell suspension was left for 30 min at room temperature before analysis. The excitation and emission wavelengths were measured at 350 and 420 nm, respectively, with slit widths of 5 nm (Synergy H1 microplate reader, Biotek-Agilent, Winooski, VT, USA). Experiments were performed by dissolving NPN in acetone (500 μM) and added to a 1 mL cell suspension to a final 10 μM concentration. Thereafter, an initial baseline was recorded from the first 30 s of NPN addition. The increase NPN fluorescence intensity was recorded over 360 s upon the addition of colistin. The average of the baseline was taken over 30 s and subtracted from the highest response of each colistin antibiotic concentration.

### 2.8. Atomic Force Microscopy

AFM studies were carried out as detailed earlier [25] with slight modification. AFM investigated the morphology and surface properties of colistin-resistant and susceptible *E. coli* and *A. veronii* cell suspensions. The experiment was done by harvesting mid-logarithmic cells of *E. coli* and *A. veronii* from CaMHB by centrifugation at 6000× *g* (rpm) for 5 min at 25 °C. The bacterial suspensions were washed 6 times in Milli-Q water and resuspended to obtain 1 × 10^8^ CFU/mL. To subject the sample to AFM imaging, a clean glass slide was poured with 5–10 μL drop of bacterial suspension and allowed to air dry. The effect of colistin treatment was examined by adding 2 µg/mL colistin to bacterial cultures of *E. coli* and *A. veronii*. The treated cultures were incubated at 37 °C in an incubator shaker for 30 min; this time duration ensured cells were left for AFM imaging after rapid concentration-dependent bactericidal activity [26]. Upon centrifugation and washing, a 5–10 μL drop of bacterial suspension was deposited on a glass slide allowed to air dry for AFM imaging by Varian FT-Raman and Varian 600 UMA, Lake Forest, CA, USA and the results were analyzed with WITec Project Data Analysis Software, WITec Instruments Corp. Ulm, Germany.

### 2.9. Zeta Potential Measurements

The bacterial surface was washed twice with Milli-Q water and resuspended in the same Milli-Q water to obtain 10^8^ CFU/mL. Suspensions were filled in disposable clear folded capillary Zeta cells (Analyzer Malvern Zeta sizer Nano ZS, Malvern, Worcestershire, UK). The electrophoretic mobility (EPM) of bacterial cells was recorded at 25 °C and 150 V using a Zeta potential analyzer. EPMs were further converted to Zeta potentials with the help of Helmholtz–Smoluchowski theory. Triplicate experiments were performed on three separately prepared samples as previously described [27]

### 2.10. ^1^HNMR Analysis of Colistin Binding with LPS Aggregates

Colistin antibiotic was dissolved in 0.5 mL of D_2_O (pH 4.0) to a final concentration of 0.1 mM. The binding of colistin to LPS was examined by line broadening in the NMR spectra. The experiment was carried by adding small volume aliquots of LPS solution to increasing colistin concentration. The one-dimensional proton NMR (^1^H NMR) spectra was recorded on 500 MHz Nuclear Magnetic Resonance (CP-MAS—Bruker Avance III, Fallanden, Switzerland) operating at 20 °C. 3-(trimethyl-silyl)-1-propane sulfonic acid sodium salt was used as a reference for the chemical shift. Mest RC software was used for the data processing and analysis as previously described [28].

## 3. Results

### 3.1. MIC Determination and LPS Extraction by SDS-PAGE

Minimum inhibitory concentration (MIC) breakpoints of colistin against susceptible and resistant strains were interpreted according to the European Committee on Antimicrobial Susceptibility Testing (EUCAST) guidelines. The susceptible strains displayed a ~256-fold greater susceptibility to colistin compared with the resistant strains (Table 1). The extracted LPS were run on SDS-PAGE, silver-stained and visualized under a gel documentation system (BIO-RAD, Hercules, CA, USA).

### 3.2. Deoxycholate Permeability Assay

**Figure 1:**
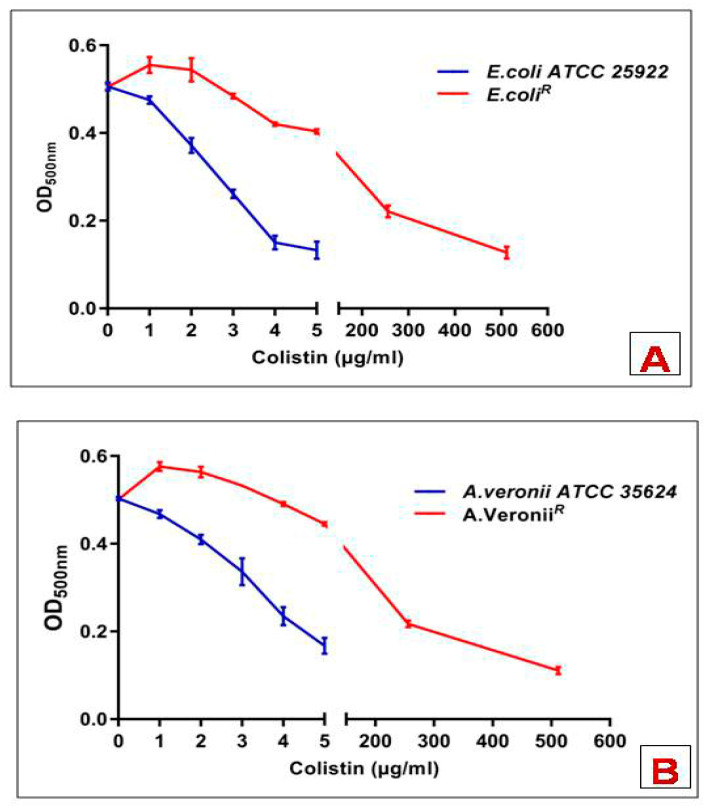
Deoxycholate-induced cell lysis of bacterial strains in response to increasing concentrations of colistin (**A**) *E. coli*; (**B**) *A. veronii.* Data points are the mean ± SD of three independent measurements.

Following incubation with deoxycholate or colistin alone, bacterial cell suspension had a negligible effect on the OD. Whereas deoxycholate-containing cell suspension upon addition with colistin caused a decline in the OD, this might be possibly because of deoxycholate-mediated cell lysis. All the experiments were performed in triplicates. Susceptible strains showed a sharp decrease in OD upon exposure of different concentrations of colistin to deoxycholate-mediated time-course reaction. However, resistant strains showed lysis only at colistin concentration ≥256 µg/mL (Figure 1).

### 3.3. Lysozyme Sensitivity Assay

**Figure 2:**
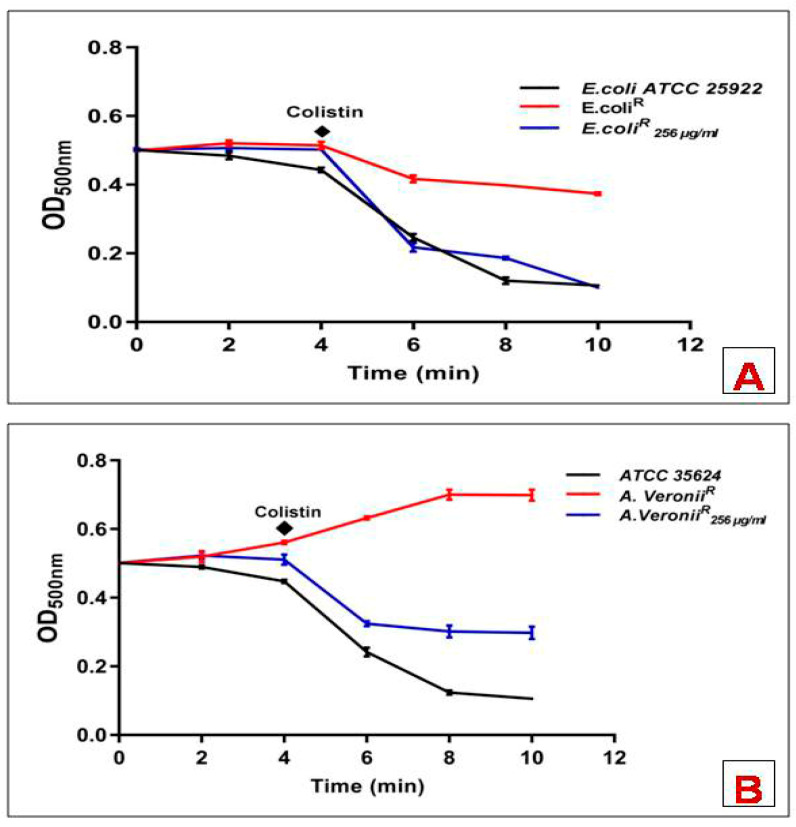
Lysozyme induced changes in OD_500_ of mid-logarithmic phase cell suspensions of bacterial strains exposed to colistin (**A**) *E. Coli*; (**B**) *A. veronii.* Data points are the mean ± SD of three independent measurements.

Following incubation with lysozyme or colistin alone, whole-bacterial cell suspension showed an insignificant effect on the OD. However, reduced OD was recorded in cell suspension upon adding colistin in the presence of lysozyme, which could be described as elevated lysozyme-mediated cell lysis. All the experiments were performed in triplicates. For the susceptible strains, lysozyme time-course reactions showed a rapid decline in OD following the exposure of colistin, which suggested an immediate disruption of the outer cell membrane. However, resistant strains showed lysis only at colistin concentration ≥256 µg/mL (Figure 2).

### 3.4. N-phenyl-1-Naphthylamine Uptake Assay

**Figure 3:**
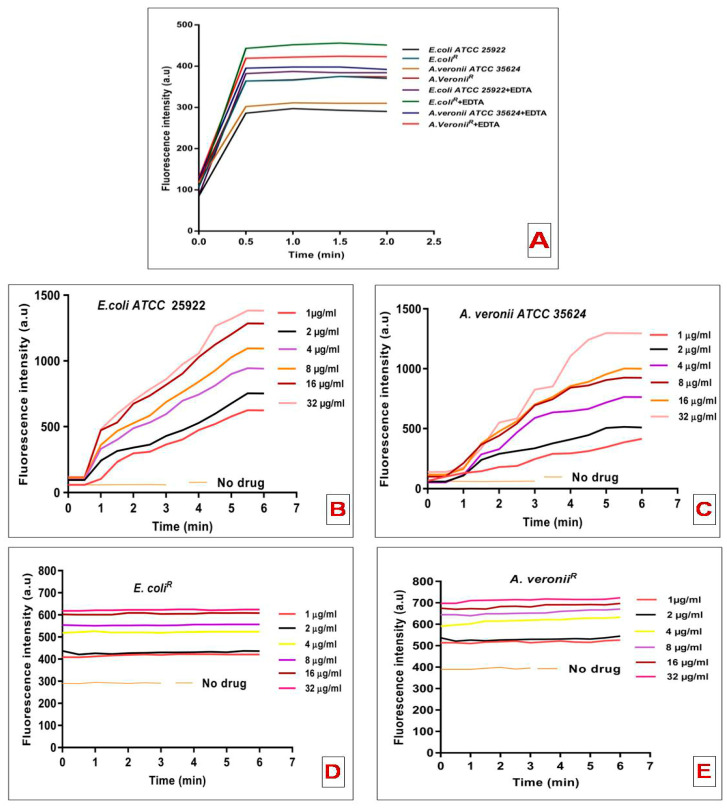
NPN uptake kinetics of mid-logarithmic phase of *E. coli* and *A. veronii* cell suspension (**A**); Effect of increasing concentrations of colistin on the uptake of NPN into the outer membrane of *E. coli ATCC 25922* (**B**); *A. veronii ATCC 35624* (**C**); *E. coli*^R^ (**D**) and *A. veronii*^R^ (**E**).

The permeabilizing activity of colistin was determined by NPN uptake to the outer cell membrane of bacterial strains. The increase in fluorescence intensity was observed following the addition of a fixed concentration of NPN to *E. coli* and A. veronii cell suspensions without colistin antibiotic (Figure 3A). Higher basal levels of NPN uptake were observed for the resistant strain than the susceptible strain; showed a more permeable outer cell membrane structure. To validate the hypothesis that the higher NPN uptake might be a consequence of lower LPS-to-LPS interactions by weaker divalent cations binding, the effect of EDTA, a chelating agent, on NPN uptake was evaluated. To verify this hypothesis, sequestration of divalent cations on the membrane by EDTA should have minimal effect on NPN uptake. An increase in the NPN entry kinetics upon addition of EDTA was observed for both the resistant strains (Figure 3A). The effect of EDTA on NPN permeability was insignificant for the resistant strains, which conferred that outer membrane integrity is hardly dependent on the stabilization of divalent cations. Figure 3B,C represents the NPN kinetics for the fluorescence variation recorded upon addition of NPN and fixed doses of colistin concentrations to *E. coli* and A. veronii cell suspensions. In the susceptible strains, colistin-induced NPN uptake was quick and reached a plateau level (Figure 3B,C). However, resistant strains did not produce any change in NPN basal level uptake even at the higher colistin (32 μg/mL) concentration (Figure 3D,E).

### 3.5. Atomic Force Microscopy

**Figure 4:**
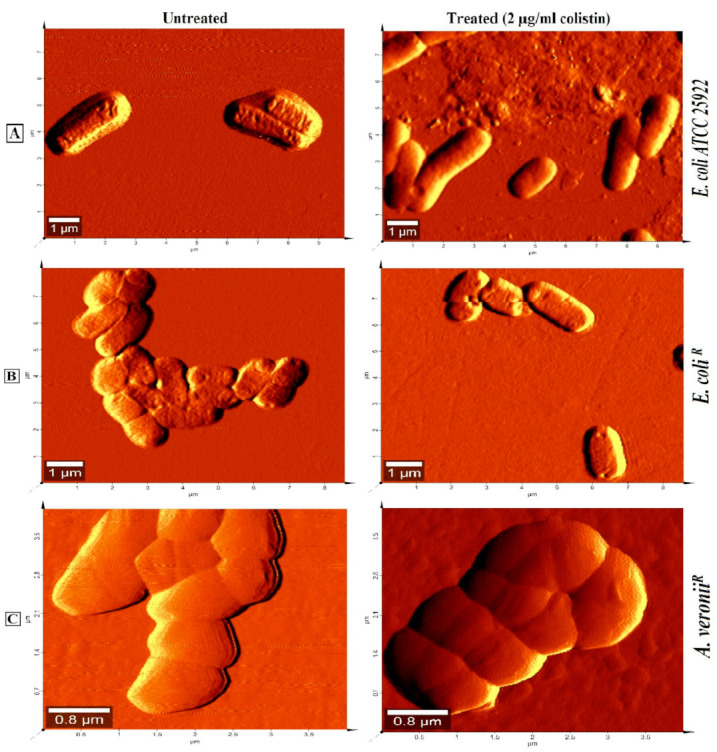
Atomic force microscopy images in dimensions (μm) of mid-logarithmic phase colistin susceptible and resistant cells treated with 2 µg/mL colistin for 20 min (**A**); *E. coli* ATCC 25922 (**B**); *E. coli*; (**C**) *A. veronii^R^*.

AFM was used to differentiate the morphology of treated and untreated susceptible as well as resistant strains. The study revealed smooth, featherless and deformed membrane structure in treated sensitive cells (Figure 4A). However, treated resistant strains exhibited lesser smoothness even at higher colistin (256 µg/mL) concentration (Figure 4B,C).

### 3.6. Zeta Potential Measurements 

**Figure 5:**
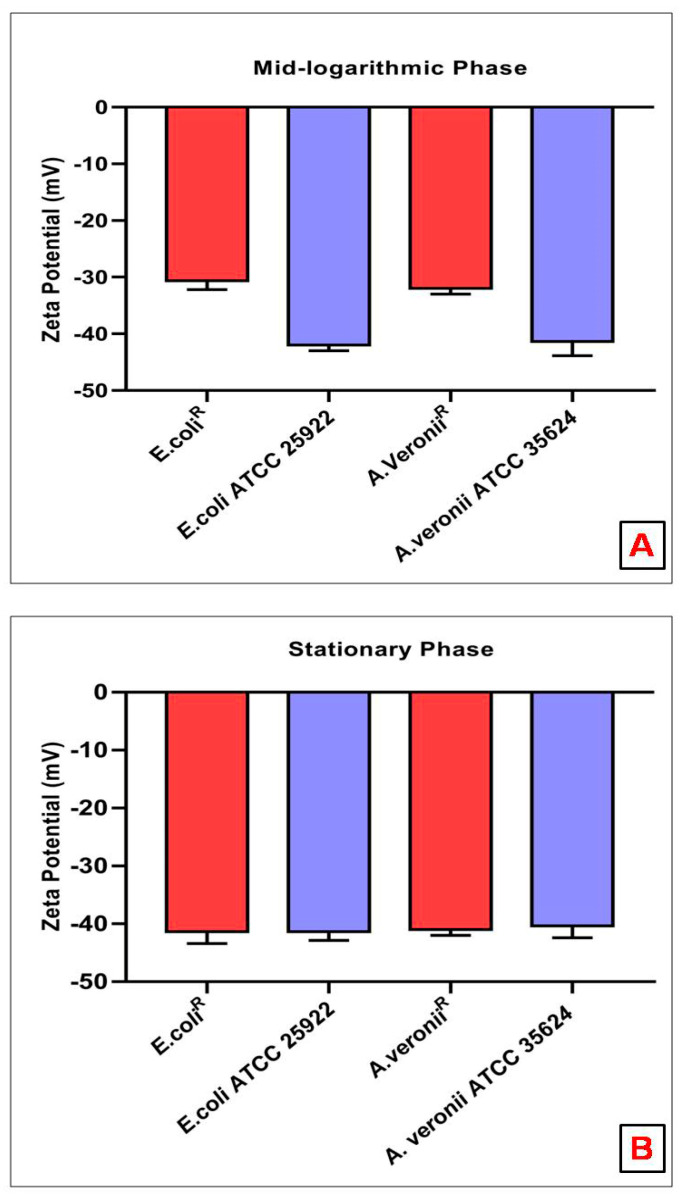
Zeta potential (mean ± SD) of colistin-susceptible and resistant *E. coli* and *A. veronii* at (**A**) mid-logarithmic Phase; (**B**) stationary phase.

The Zeta potential was recorded for mid-logarithmic and stationary phase bacterial cell suspensions. A higher negative charge (*E. coli* 25922 −42.0 ± 0.6, *A. veronii* 35624 −41.3 ± 1.4) was observed for mid-logarithmic phase susceptible bacterial cells than the mid-logarithmic phase resistant bacterial cells (*E. coli^R^* −30.4 ± 0.9, *A. veronii^R^* −32.0 ± 0.6) (Figure 5A). However, Zeta potential measurement was not statistically significant in stationary phase cells of each strain (Susceptible: *E. coli* 25922 −41.3 ± 0.9, *A. veronii* 35624 −40.33 ± 1.2; resistant: *E. coli*^R^ −41.3 ± 1.2, *A. veronii*^R^ −41.0 ± 0.6) (Figure 5B).

### 3.7. ^1^HNMR Analysis of Colistin Binding with LPS Aggregates

**Figure 6:**
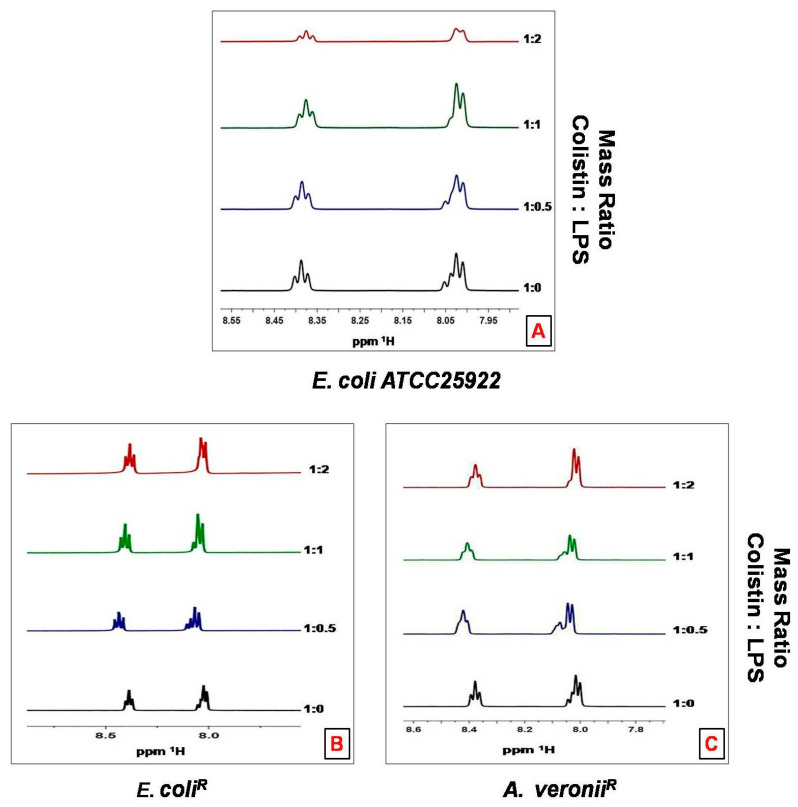
500 MHz ^1^H NMR spectrum of the amide region of colistin in 10% D2O pH 4.0 titrated with LPS aggregates. The ratio of LPS to colistin is indicated on the right ordinate (**A**) *E. coli ATCC 25922*; (**B**) *E. coli^R^*; (**C**) *A. veronii^R^*.

Titration of LPS aggregates into a solution of colistin produces a line-broadening of the colistin resonances with binding to LPS, for simplicity, only the amide region is shown in NMR spectra (Figure 6A). On the contrary, line broadening was not recorded for the resistant strains, even at the highest colistin: LPS mass ratio (Figure 6B,C).

## 4. Discussion

Resistance to colistin, one of the last-line antibiotics to tackle infections from Gram-negative bacteria has provided the impulsion to elucidate resistance mechanisms employed by pathogenic bacteria. It has been reported that cationic antimicrobial peptide resistance in bacteria was assisted by the thickening of capsular polysaccharide layers [29]. It has been suggested that the bactericidal action of colistin on bacteria involves a couple of interactions with the surface as well as intracellular components [30]. This study employed various biophysical techniques to elucidate the interactions between colistin and outer membrane surfaces from susceptible and resistant strains of *E. coli* and *A. veronii*. It was observed that susceptible strains depicted higher sensitivity to the lytic action of lysozyme and sodium deoxycholate on the inner membrane and periplasmic peptidoglycan, respectively, in comparison to the resistant strains that followed previously studied colistin-resistant and susceptible *Salmonella typhimurium* bacteria [23]. These results may be imputed to a greater tendency of colistin to adversely affect the outer membrane of the susceptible strain, resulting in the access of these agents to disrupt internal structures. The permeability pattern was further elucidated by a hydrophobic probe i.e., NPN, known to exhibit increased fluorescence in a hydrophobic environment [31]. NPN uptake kinetics were significantly increased in resistant strains in comparison to susceptible strains, even in the absence of colistin treatment [32]. Comparative studies revealed that resistant strains constitute a more penetrable, less dense outer membrane structure. Moreover, EDTA, a chelating agent, promotes the sequestering of divalent cations that link adjacent outer membrane molecules in Gram-negative outer leaflets, did not affect NPN uptake in colistin-resistant strains [32]. Colistin-induced NPN uptake in the susceptible strain was rapid and reached a plateau level in a relatively lesser time. On the contrary, change in the basal level of NPN uptake was not observed upon addition of colistin to resistant strains even at the highest concentrations. Similar results were reported in other resistant strains of *Klebsiella pneumonia* [28]. Moreover, AFM results revealed smooth, featherless and deformed membrane structure in treated sensitive cells. However, treated resistant strains exhibited less smoothness even at higher colistin concentration (256 µg/mL). These findings are in consonance with studies on other Gram-negative bacteria at different growth phases and after colistin exposure [25]. Outer membrane electrostatic variations recorded by Zeta potential deduced that stationary phase cells of each bacterial strain did not witness any significant change in surface charge. However, the mid-logarithmic phase susceptible bacterial cells displayed a higher negative charge than the resistant cells. The magnitude of Zeta potential observed in mid-logarithmic phase *E. coli* and *A. veronii* strains in our study is in corroboration with the reports on Zeta potential measurement of other Gram-negative species under similar conditions [28,33,34,35,36].The less-negative Zeta potential expressed by colistin-resistant cells at mid-logarithmic phase is mainly because of changes in the structure and composition of the outer membrane. A similar study carried out by Soon et al., justified our analysis that colistin-resistant cells have a greater tendency to clump in small clusters or chains in contrast with colistin susceptible cells [25]. It has also been reported that colloid aggregate stability is increased with particle carrying a lower magnitude of charge resulting in reduced electrostatic repulsion [33].The charge shielding esterification of phosphates on lipid A with 2-aminoethanolor 4-amino-4-deoxy-L-arabinose has previously been reported in colistin-resistant bacterial strains of *E. coli* [37], *Pseudomonas aeruginosa* [38], *Salmonella typhimurium* [39] and *Yersinia pestis* [40]. Thereafter, the interaction of colistin with LPS aggregates was studied by ^1^H NMR measurements to evaluate the line broadening of amide regions of colistin in resistant and susceptible strains. Line broadening was observed in the titration of LPS aggregates and different concentrations of colistin, for better understanding, only amide regions were observed. Interestingly, in the case of colistin susceptible cells, line broadening was recorded with increasing colistin: LPS mass ratio. However, colistin-resistant cells did not show line broadening even at the highest mass ratio; these observations are in line with an earlier report on other Gram-negative bacteria [28]. Taken together our results propose that the colistin-resistant strains carry a more permeable outer membrane with less negative charge as compared to the susceptible strains. Reportedly, modulation on the outer membrane component results in the emergence of resistance against colistin antibiotic among Gram-negative bacteria. This is evident from the fact that colistin resistance in Gram-negative bacteria involves modification on lipid A. Moreover, the abolition of divalent cations that link adjacent LPS molecules affects the packing of the lipid A membrane [41]. The outer membrane of resistant bacteria should increase the access of the surface to colistin action by the hydrophobic domain of the antibiotic. Previous studies reported that the interaction of colistin with outer membrane require an initial electrostatic attraction that facilitates the sufficient stabilization of the complex to initiate the second-stage interaction between hydrophobic regions of Gram-negative bacteria [30,42,43]. Therefore, the implication is of an outer membrane resistance mechanism to nullify the important electrostatic interactions which eventually increase the membrane fluidity results in the vulnerability to the colistin attack. In summary, our study showed that the colistin resistance mechanisms employed by *E. coli* and *A. veronii* renders complex outer surface membrane changes on the bacterial surface that together block the capability of the colistin antibiotic to constitute critical electrostatic interactions with the bacteria and inhibit bactericidal activity.

## 5. Conclusions

This study added further evidence to colistin-resistance mechanisms that contribute to outer membrane surface changes in Gram-negative bacteria. These surface modifications obstruct colistin to establish critical electrostatic contacts with the outer membrane surface that leads to bactericidal activity. This study highlights the first report of the evaluation of surface changes in colistin-resistant strains of *E. coli* and *A. veronii*. This study also reports surface alterations associated with the resistance mechanisms employed by the plasmid-mediated resistance (*mcr*-1) gene. However, there is still a lack of knowledge regarding colistin binding and initiating bactericidal activity. As new subtypes of plasmid-mediated resistance genes have emerged that express outer membrane modification, studies need to be done to trace all possible mechanisms of colistin resistance.

## Figures and Tables

**Table 1:** MICs (μg/mL) of colistin tested against susceptible and resistant strains.

S. No.	Strains	MIC (µg/mL)
1	*E. coli* (*ATCC 25922*)	1
2	*A. veronii* (*ATCC 35624*)	0.5
3	*E. coli^R^* (*AF15*)	256
4	*A. veronii^R^* (*AF6*)	256

## Data Availability

Not applicable.
